# The discussion of t(1;17)(p11;q21) translocation in acute promyelocytic leukemia patient on molecular remission

**DOI:** 10.1002/ccr3.1108

**Published:** 2017-08-17

**Authors:** Yannan Jia, Chengwen Li, Jiawei Zhao, Yang Song, Juan Wang, Yingchang Mi

**Affiliations:** ^1^ State Key Laboratory of Experimental Hematology Department of Clinical Hematology, and Department of Hematopathology Institute of Hematology and Blood Diseases Hospital Chinese Academy of Medical Sciences & Peking Union Medical College Tianjin China; ^2^ Department of Clinical Hematology Cangzhou Central Hospital Hebei China

**Keywords:** Acute promyelocytic leukemia, karyotype, t(1;17)(p11;q21)

## Abstract

Some chromosomal aberrations emerging in the course of treatment are probably not related to disease progression, but attribute to the germline alteration. Therefore, the dynamic genetic tests should be performed during the whole treatment process, which is significantly essential for efficacy evaluation and treatment decision‐ making.

## Introduction

Acute promyelocytic leukemia (APL) is a special disease entity of acute myeloid leukemia (AML), comprising about 5–8% of total AML cases. The 2001 revision of the World Health Organization (WHO) classification has recognized APL as a separate subtype of AML with recurrent cytogenetic abnormality characterized by t(15;17)(q22;q21) chromosomal translocation. The chromosomal rearrangements of 17q21 leading to fusion of the gene encoding retinoic acid receptor alpha (RAR*α*) to a number of alternative partner genes (X), the most frequent of which are PML (>95%, PML‐RAR*α* fusion gene) 1. Although the title of this subtype is not identical in 2001, 2008, and 2016 version of WHO classification, they consistently reserved PML‐RAR*α* fusion gene positive as a basic criteria.

Cases without classic PML‐RAR*α* fusion gene but still morphologically compatible with a diagnosis of APL have been found to present with other rare cytogenetic abnormities which is also involved with RAR*α* gene, while PML gene is replaced by other genes at different chromosomes, subsequently forming a series of chimeric genes with distinct function 2, 3, 4, 5 (Table 1). Here, we present a rare case which involved with chromosome 17q21, while no RAR*α* gene involvement. The details are described as follows.

**Table 1:** Chromosome translocations and fusion gene in acute promyelocytic leukemia

Cytogenetics	Fusion proteins
t(15;17)(q22;q21)	PML/RARA
t(11;17)(q23;q21)	ZBTB16/RARA
t(5;17)(q35;q21)	NPM/RARA
t(11;17)(q13;q21)	NUMA/RARA
der(17)	STAT5B/RARA
der(17)	PRKR1a/RARA
t(X;17)(p11;q12)	BCOR/RARA
t(4;17)(q12;q21)	FIP1L1/RARA
t(3;17)(q26;q21)	TBLR1‐RARa

## Case Presentation

**Figure 1:**
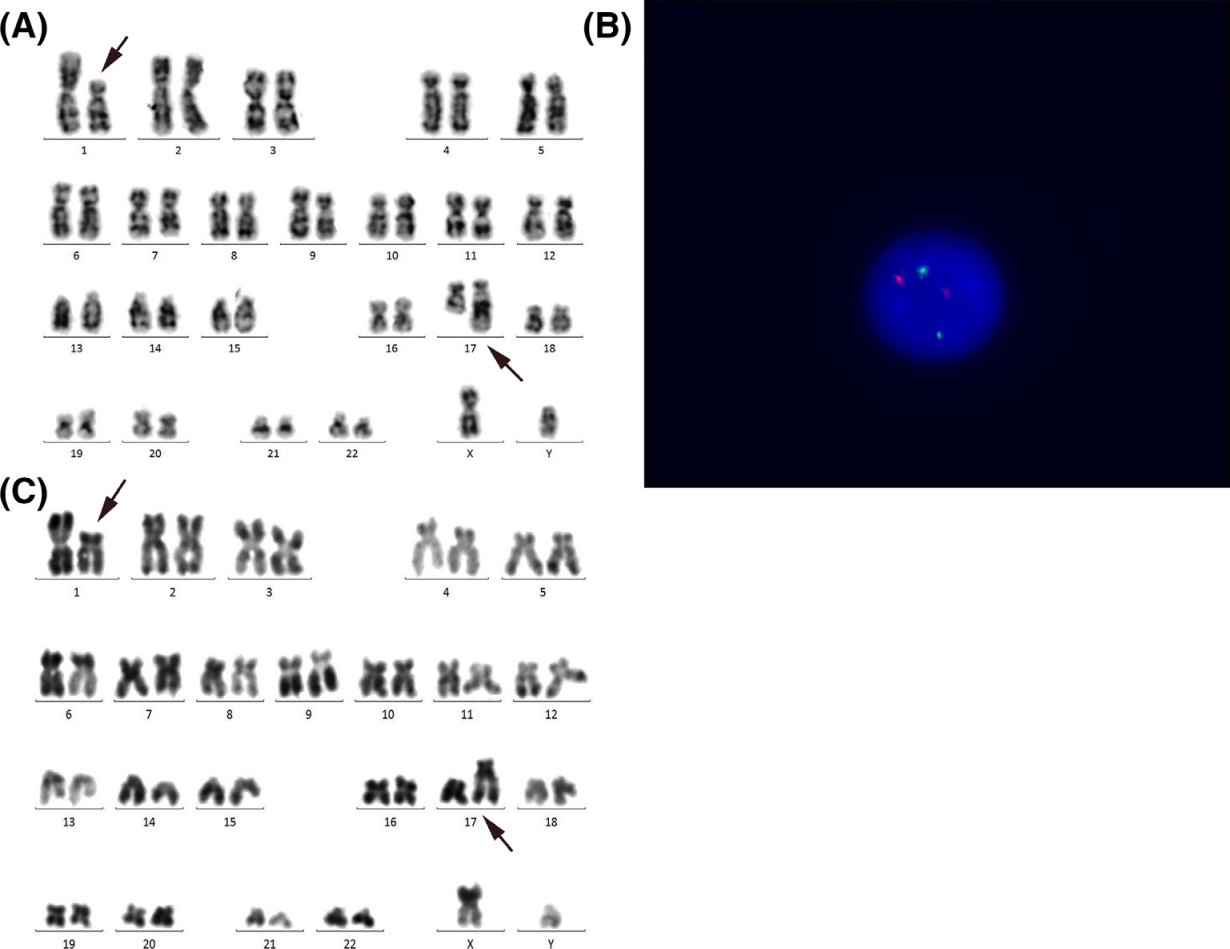
Cytogenetic analysis of BM and peripheral sample of the APL case with t(1;17)(p11;q21) translocation. (A) Karyotype analysis of BM sample: 46,XY, t(1;17)(p11;q21)[20]. (B) FISH analysis. PMLprobe (orange signal) and RAR *α* probe (green signal) were used. No PML‐RAR *α* was detected. (C) Karyotype analysis of peripheral blood cells 46,XY, t(1;17)(p11;q21).

A 19‐year‐old young man was diagnosed and treated as acute promyelocytic leukemia (APL) at other hospital in 2012. Reverse transcription‐polymerase chain reaction (RT‐PCR) showed PML‐RAR*α* positive, while cytogenetic analysis was unknown at the time of diagnosis. Patient achieved complete remission (CR) after all‐trans retinoic acid (ATRA) and arsenic trioxide (ATO) induction treatment, and then continually managed with chemotherapy, all‐trans retinoic acid (ATRA) and arsenic trioxide treatment. The whole process lasted more than 1 year. He came to our hospital for an examination on October, 2014. His past history, personal history, and family history were unremarkable. Physical examination: No jaundice, bleeding spots or Bruise on skin. Superficial lymph nodes were not found enlarged. Bilateral eyelid conjunctiva was not cadaverous, pharynx was not congestive, tonsils were not enlarged. Sternum was without tenderness. The breath sounds clear without rales. The heart sounds normal with regular rhythm. The liver and spleen were not palpable. No edema in the lower extremities. Laboratory tests revealed: WBC 1.7 × 10^9^/L, RBC 5.66 × 10^12^/L, Hb 162 g/L and platelet count (PLT) 167 × 10^9^/L. Bone marrow aspiration, conventional cytogenetic analysis and PML‐RAR*α* (RT‐PCR) were all performed. Bone marrow (BM) smear showed hypercellularity with approximately 74% hypergranular promyelocytes and Auer body, which was considered as a recurrence manifestation of APL after treatment. PML‐RAR*α* level was 54% and karyotype analysis revealed 46,XY,t(1;17)(p11;q21)[20]. He received re‐induction with ATO plus Daunorubicin (DNR) and achieved CR2. Then four courses of consolidation treatment were consecutively administered to this patient as follows: ATO+Mitoxantrone (MTZ), ATO+MTZ, ATO+MTZ, ATO+DNR. After the second consolidation course, the PML‐RAR*α* fusion gene could not be detected by RT‐PCR test and remained negative since then. Then the patient remained on maintenance therapy based on ATO and ATRA. The last two re‐examination (the last time was on 14 February 2017) results of karyotype analysis both showed 46,XY,t(1;17)(p11;q21)[20] (Fig. 1A), whereas the PML‐RAR*α* level which detected simultaneously was 0%.

To explore the real meaning of this case, we firstly examined the PML‐RAR*α* fusion gene (interphase cell from bone marrow sample) using fluorescence in situ hybridization (FISH) study, which also showed negative result, implying that there was no RAR*α* gene involvement (Fig. 1B). Then we performed karyotype analysis of lymphocytes isolated from the patient's peripheral blood cells and got the same result. Based on these clearly evidences, we confirmed that the chromosomal aberration emerged in this patient attributed to the germline alteration, instead of a disease‐related alteration, and could not be regarded as an indicator to adjust the treatment strategy. At present, the patient is still at the follow‐ up.

## Discussion

The majority of acute promyelocytic leukemia (APL) cases have an identifiable t(15;17)(q22;q12‐21) chromosomal aberration, only a few present with complex, cryptic or variant translocations, almost all involve RAR*α* gene. No APL variants with PML involvement alone have been identified to date 6. The dynamic genetic tests (including both cytogenetic analysis and fusion gene examination) 7 should be performed during the whole treatment process, which is significantly important for efficacy evaluation and treatment decision making.

However, we should recognize that clinical cases have their own unique characteristics. Patients suffered from AML (especially those with recurrent cytogenetic abnormality) or chronic myelogenous leukemia (CML) (and some other types of hematological malignancy) can either present with typical cytogenetic abnormality or exhibit with other abnormality such as complex (accompanied with additional aberration) or variant translocations. How to make accurate evaluation for these atypical alterations and additional aberrations is of great significant for disease prognostic stratification, efficacy assessment, and treatment adjustment. Disease‐related cytogenetic abnormality would be restored (achieving cytogenetic response) along with the recovery of disease, at least the number of abnormal mitotic figures would greatly reduce. Normally, the aberrant chromosome alterations would be restored after treatment especially when the specific fusion genes had become negative (at least the involved chromosome alterations related to the fusion genes should disappear).

This patient carried an PML‐RAR*α* fusion gene which was confirmed on diagnosis and replase. Instead of presenting a classic t(15;17) abnormity, he showed an rare 46,XY,t(1;17)(p11;q21)[20] translocation (Fig. 1A). Particularly, the chromosome 17 where RAR*α* gene located was involved. The PML‐RAR*α* fusion gene was turned negative after treatment, while all of mitotic figures examined for karyotype analysis was still presented with t(1;17)(p11;q21). To explain this phenomenon, we first tried to detect the signal of PML/RAR*α* fusion gene derived from bone marrow using FISH, which proved that no RAR*α* gene was involved. Such result highly suggested that this kind of abnormity was probably not related to disease progression (germline alteration ?). To verity this possibility, we isolated the lymphocytes from this patient's peripheral blood cells and analyzed karyotype of lymphocyte from this patient's which showed the same chromosomal alterations (Fig. 1C). Therefore, we can conclude that the chromosomal aberration emerged in this patient attributed to the germline alteration, rather than APL related. So, we decided to stop maintenance treatment and reexamine regularly according to the plan.

## Conflict of Interest

The authors declare no potential conflict of interests.

## Authorship

YM: involved in conceptualization. YJ, CL, JZ, YS, JW: involved in data curation. CL: involved in formal analysis. JW: involved in investigation. YM: involved in methodology. YM: involved in supervision. YJ: involved in Writing—original draft. YM: involved in writing—review and editing.
